# Time and dose-dependent effects of *Labisia pumila* on the bone strength of postmenopausal osteoporosis rat model

**DOI:** 10.1186/s12906-015-0567-x

**Published:** 2015-03-12

**Authors:** Nadia Mohd Effendy, Shahrum Abdullah, Mohd Faridz Mod Yunoh, Ahmad Nazrun Shuid

**Affiliations:** Department of Pharmacology, Faculty of Medicine, Universiti Kebangsaan Malaysia, Jalan Raja Muda Abd Aziz, 50300 KL Kuala Lumpur, Malaysia; Department of Mechanical and Materials Engineering, Faculty of Engineering, Universiti Kebangsaan Malaysia, Kuala Lumpur, Malaysia

**Keywords:** Osteoporosis, Postmenopausal, Estrogen, Bone strength, Biomechanical

## Abstract

**Background:**

Post-menopausal osteoporosis has long been treated and prevented by estrogen replacement therapy (ERT). Despite its effectiveness, ERT is associated with serious adverse effects. *Labisia pumila var. alata* (LP) is a herb with potential as an alternative agent to ERT due to its phytoestrogenic, antioxidative and anti-inflammatory effects on bone. This study aimed to determine the effects of LP supplementation on bone biomechanical strength of postmenopausal osteoporosis rat model.

**Methods:**

Ninety-six female Sprague–Dawley rats aged 4 to 5 months old were randomly divided into six groups; six rats in the baseline group (BL) and eighteen rats in each group of; Sham- operated (Sham), ovariectomised control (OVXC) and ovariectomised with daily oral gavages of Premarin at 64.5 μg/kg (ERT), LP at 20 mg/kg (LP20) and LP at 100 mg/kg (LP100) respectively. These groups were subdivided into three, six and nine weeks of treatment periods. Rats in BL group were euthanized before the start of the study, while other rats were euthanized after completion of their treatments. Femora were dissected out for biomechanical strength analysis using Instron Universal Model 5848 Micro Tester.

**Results:**

OVXC group showed deterioration in the bone biomechanical strength with time. Both ERT and LP supplemented rats showed improvements in bone strength parameters such as maximum load, displacement, stiffness, stress, and Young Modulus. The most improved bone strength was seen in rats given LP at the dose of 100 mg/kg for nine weeks.

**Conclusion:**

LP supplementation at 100 mg/kg was more effective than ERT in reversing ovariectomy-induced bone biomechanical changes.

## Background

Osteoporosis is considered as a serious public health concern due to its increasing prevalence worldwide. It is a skeletal disease characterized by low bone mass and microarchitectural deterioration with a consequent increase in bone fragility and susceptibility to fracture [1]. The main types of osteoporotic fractures are hip, wrist and vertebral fracture. Hip fracture is the most common form of osteoporotic fractures and is the most serious complication of osteoporosis. The hip fracture incidence has been reported to increase worldwide during the last five decades [2,3]. Women are more susceptible to hip fractures due to their lower bone density compared to men. The lifetime risk of hip fracture was 17.5% in women and 6% in men [4]. The current total number of women sustaining a hip fracture is estimated to be one million annually [5] and the total number of hip fractures are expected to surpass 6 million by the year 2050 [6]. Women during the senescence years are mostly affected due to estrogen deficiency due to menopause or bilateral ovariectomy. This condition leads to accelerated bone loss and promotes the development of postmenopausal osteoporosis [7,8].

Estrogen plays an important role in bone remodelling via direct effects on estrogen receptors (ERα and ERβ) located on bone cells. Activation of estrogen-receptor complex will stimulate osteoblast differentiation and simultaneously suppress osteoclast activity [9]. The activation of estrogen-receptor complex will downregulate Receptor activator of nuclear factor kappa-B ligand (RANKL), which is crucial for osteoclast formation. Estrogen will also stimulate the production of an osteoclastogenesis inhibitory factor known as osteoprotegrin (OPG), which will suppress osteoclastic activity. Oppositely, estrogen deficiency will lead to the upregulation of cytokines such as interleukin-1 (IL-1), IL-6, macrophage colony-stimulating factor (M-CSF) and tumor necrosis factor (TNF-α) [10]. These inflammatory cytokines will induce osteoclast differentiation and inhibit its apoptosis. Based on the aforementioned bone protective effects of estrogen, it is obvious that estrogen deficiency in postmenopausal women will result in bone loss particularly cancellous bone on the endosteal surface, which is an active site for bone growth and remodeling [11,12]. Bone formation activity is unable to keep pace with bone resorption, resulting in bone loss and fracture risk.

Since estrogen protects against bone loss, its deficiency in postmenopausal women could be replaced in the form of estrogen replacement therapy (ERT). In a longitudinal study of ERT in older postmenopausal women, ERT was found to increase the bone mineral density at both lumbar spine and proximal femur. Histological analysis also showed an increase in cancellous bone volume and wall thickness [13]. Estrogen, which can be given alone or in combination with progesterone exerted antiresorptive effects on bone cells, affecting the osteoclast activity and lifespan [14,15]. According to Komm et al. [16], one year treatment with estrogen in ovariectomised rats were sufficient to improve the biomechanical properties of trabecular bone. In another study, administration of estrogen was able to increase trabecular thickness and maintain its plate-like trabecular structures, which were correlated with improved bone strength of ovariectomised mice [17]. However, in human, prolonged use of ERT may result in long term risks which may outweigh the benefits. In a large cohort of postmenopausal women in their 60s, the use of ERT was associated with an increased risk of breast cancer, coronary heart disease, stroke and dementia [18-20].

Another form of treatment for postmenopausal osteoporosis is selective estrogen-receptor modulators (SERMs) such as raloxifene and tamoxifen. They are non-steroidal agents that bind to estrogen receptors, acting as an agonist on bone but as antagonist to other organs [21]. SERMs could positively influence the bone mineral density and strength of the lumbar spine of estrogen-deficient rats [22]. Raloxifene is the most widely used SERMs for prevention and treatment of osteoporosis. It was reported to prevent bone loss and reduce fracture risk in postmenopausal women with low bone mass [23,24]. It has been shown to improve the trabecular bone density of women with postmenopausal osteoporosis [25]. It could also improve the structural component of bone strength [26,27]. Although SERMs are known to reduce the risks of breast cancer [28], their prolonged use may result in adverse effects such as thromboembolism, uterine cancer and cataract [29-31]. Other common anti-osteoporotic agent is biphosphonates such as alendronate, risedronate and zoledronate. They are potent inhibitors of bone resorption and effective in the treatment of osteoporosis [32,33]. Their adverse effect includes abdominal pain, constipation, esophagitis and osteonecrosis of the mandible [34].

Findings linking estrogen use to several serious diseases have led to many postmenopausal women looking for other treatment options to prevent and treat postmenopausal osteoporosis. These alternative treatments should be effective and with minimal side effects. To date, some of the natural remedies used for the treatment of osteoporosis includes soy, tocotrienols, *Nigella sativa* (black seed), *Piper sarmentosum* and *Labisia pumila. Labisia pumila* (LP) is a traditional herb used widely by Asian women to treat menstrual irregularities, painful menstruation, facilitate labour, promote sexual health function and for post-partum medicine [35,36]. Besides improving women’s health, LP was also reported to be effective in treating rheumatism and sickness in the bones [37,38]. There are three known varieties of LP which are var. *pumila,* var. *alata,* and var. *lanceolata* [39,40]. In Malaysia, LP is also known by the locals as *Kacip Fatimah, Akar Fatimah, Pokok Pinggang and Belangkas Hutan* [41,42]. It has long been speculated that LP possesses phytoestrogenic properties, which may explain its therapeutic values in women’s health. Phytoestrogens are naturally-occurring plant compounds that are structurally and functionally similar to mammalian estrogens and their metabolites [43]. Most phytoestrogens such as triterpene and saponins, which are found in LP, bind to both estrogen receptors ERα and ERβ, exerting a weaker estrogenic effect compared to the natural estrogen [44]. Once bound, phytoestrogens do not act like typical estrogen agonists but rather more like selective estrogen receptor modulators (SERMs) [45]. Phytoestrogens in LP could also manipulate steroid biosynthesis by stimulating sex hormone-binding globulin (SHBG) and displacing estradiol or testosterone [46].

Phytoestrogens are often potent antioxidants and anti-inflammatory agents which may explain the effects of LP on women’s health. In a previous study by Nazrun et al. [47], it was reported that supplementation of LP at the dose of 17.5 mg/kg was able to increase bone formation marker and reduce bone resorption marker in ovariectomized rats. Another study reported an improvement in the bone structural parameters of LP-supplemented rats and was able to reverse the osteoporotic changes in ovariectomized rats [48]. Although LP was proven to exert an effective effect on bone biochemical and structural parameters, these results did not truly reflect bone strength which is an important determinant in the diagnosis of osteoporosis and assessment of fracture risk.

The gold standard of assessing risk of osteoporosis has long been the bone mineral density (BMD) assessment by dual X-ray absorptiometry (DEXA) [49-51]. Despite the sensitivity and effectiveness in assessing the risk of osteoporosis, DEXA does not take into account of bone strength, the best indicator of fracture risk. Biomechanical test is the gold standard for assessment of bone strength, which is performed by exerting a load to the bone until it fractures [52]. In animal models, bone can be dissected out and its strength tested biomechanically. However, the human bone strength could only be assessed indirectly using computer software such as finite element analysis via micro-computed tomography (Micro-CT) [53,54].

LP, being a good candidate for ERT alternative, must be able to exert and sustain its bone strengthening effects with time. To the best of our knowledge, there is no report of such parameters on LP. Therefore, this study aimed to determine the time-dependent effects of two LP doses on the bone strength of postmenopausal osteoporosis rat model.

## Methods

### Animals and treatment

96 female Sprague–Dawley rats aged 4–5 months weighing between 200-250 g were obtained from the Universiti Kebangsaan Malaysia Laboratory Animal Research Unit. The rats were housed in plastic cages at a temperature of 29 ± 3°C under natural day/night cycle. They were allowed to adjust to the new environment for a week before the study was started. They were fed with commercial food pellets (Gold Coin, Port Klang, Malaysia) and deionised water *ad libitum*. They were then randomly divided into six main groups with six rats in the baseline group (Baseline) and eighteen rats in each group of Sham-operated (Sham), ovariectomized control (OVXC), ovariectomized with estrogen Premarin at 64.5 μg/kg (ERT), ovariectomized with *Labisia pumila* at 20 mg/kg (LP20) and ovariectomized with *Labisia pumila* at 100 mg/kg (LP100) respectively. All the treatments were given daily via oral gavages. These groups were subdivided into three, six and nine weeks of treatment periods. Body weights were measured before the start of treatment and weekly until the end of the study. The study was performed according to the experimental protocol approved by Universiti Kebangsaan Malaysia Animal Ethics Committee (Ethical approval number: FP/FAR/2011/NAZRUN/30-NOVEMBER/415-NOVEMBER-2011-MAY-2012).

### *Labisia pumila var. alata* (LP) and Estrogen (ERT)

A raw powdered form of the LP was supplied by Delima Jelita Herbs (Alor Setar, Kedah). The *Labisia pumila var. alata* whole plant was ground and freeze dried into powdered form. The dried powdered LP extract was sent to Forest Research Institute Malaysia (FRIM) for phytochemical screening to detect the phytochemical constituents. Based on the phytochemical screening, LP extract used in this study was found to contain flavonoids, saponins, tannins, triterpenes and steroids. LP was dissolved in deionised water and given to the respective groups via oral gavages at doses of 20 mg/kg and 100 mg/kg rat weight daily at 9 am for 3, 6 and 9 weeks. Estrogen Premarin® (Wyeth-Ayerst, Canada) tablet containing 0.625 mg of conjugated estrogen was crushed, dissolved in deionised water and given to the respective groups via oral gavages at the dose of 64.5 μg/kg rat weight daily at 9 am for 3, 6 and 9 weeks.

### Bone sampling

Rats in the BL group were euthanized before the start of the study while the other rats were euthanized upon completion of their treatments. Femora were dissected and cleaned from all soft tissues. They were then wrapped in phosphate-buffered saline-soaked gauze and rewrapped with aluminium foil prior to storage in −70°C freezer. These bone samples were allowed to thaw at room temperature before biomechanically tested. They were also kept moist at all time during the testing procedure.

### Bone biomechanical test

**Figure 1:**
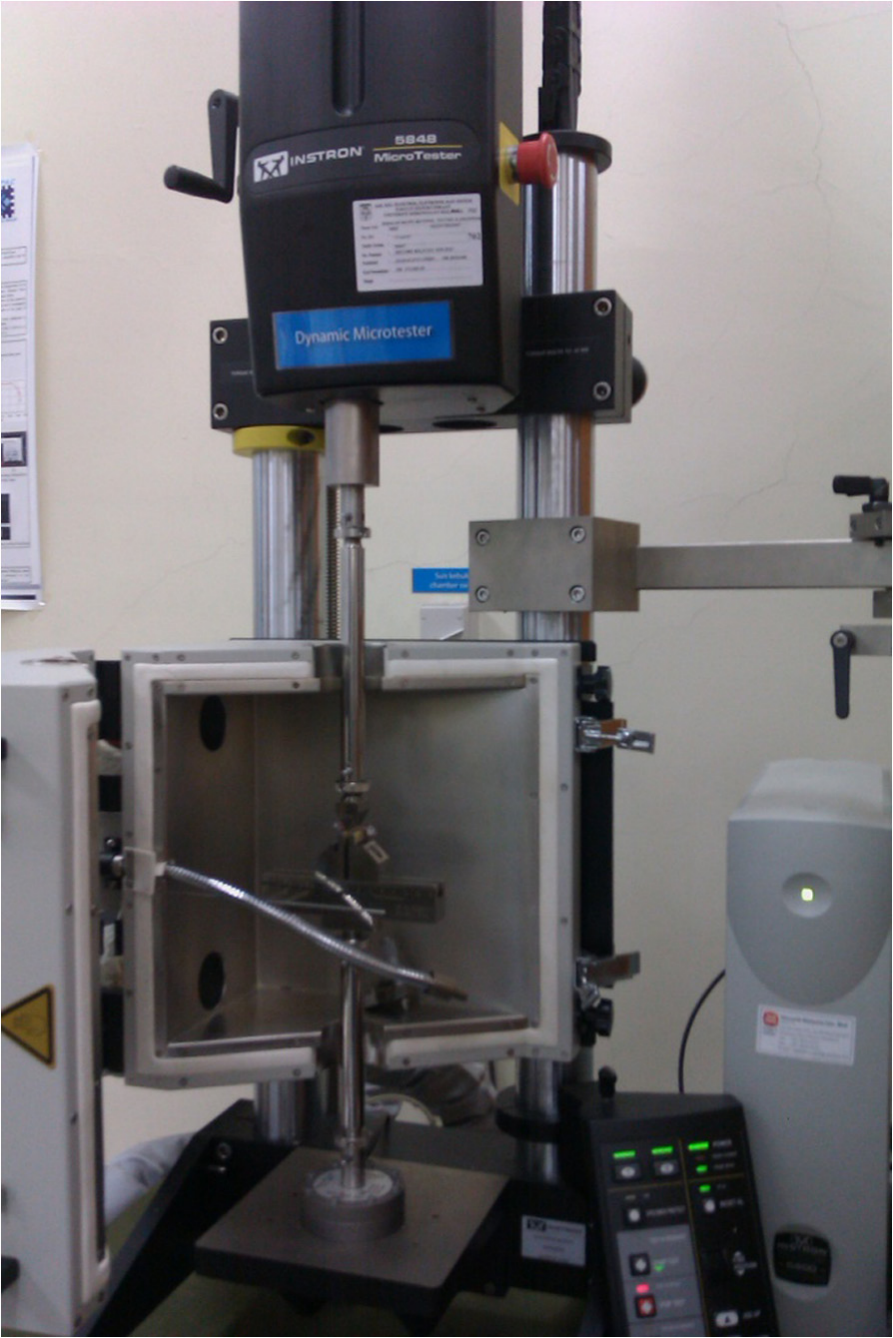
**Instron Universal Microtester.**

**Figure 2:**
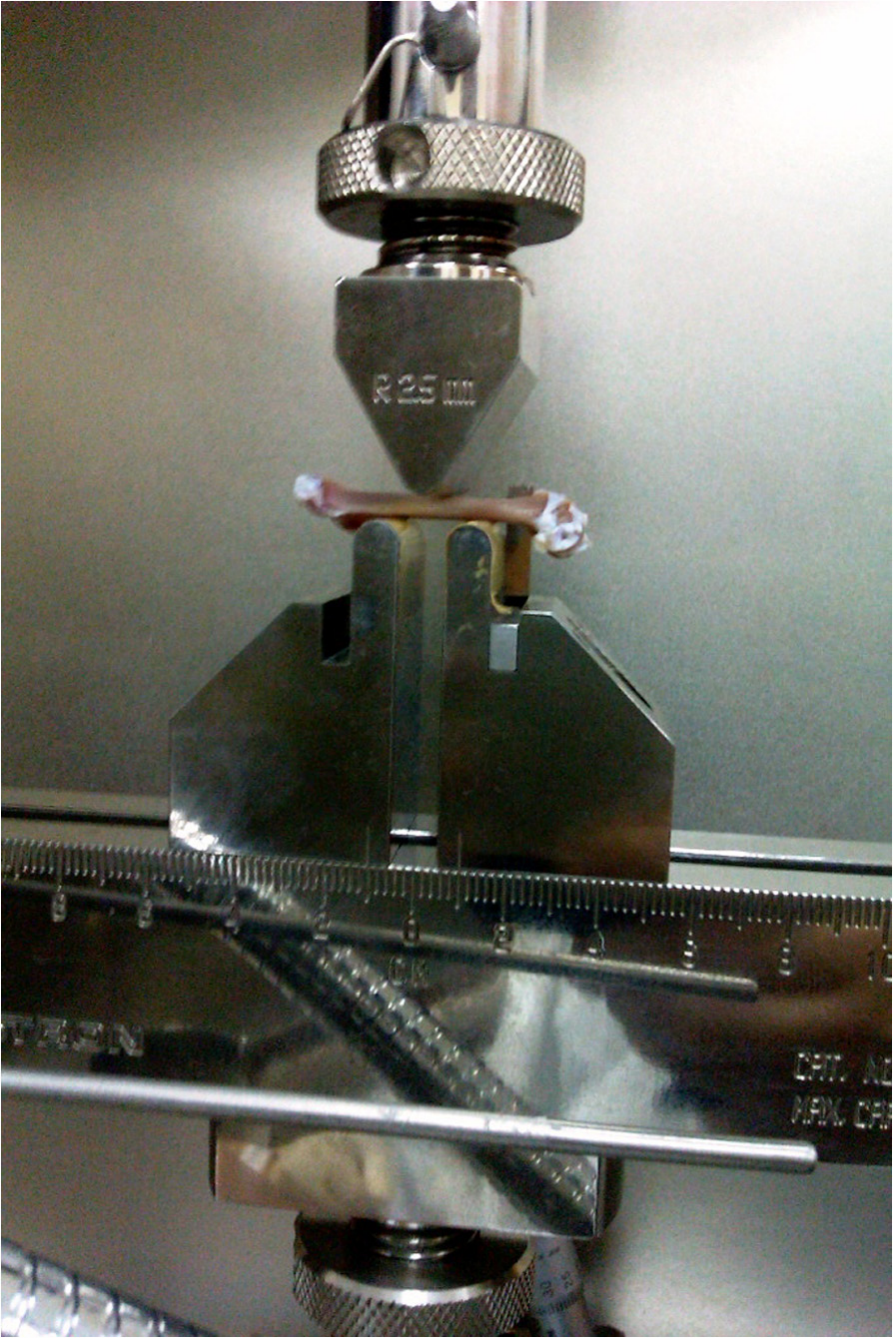
**Force applied to the midpoint of the femur.**

Biomechanical properties of the femoral bones were assessed using three-point bending test method. This test was performed using Instron Universal Testing machine (Model 5848 Microtester, Canton, USA) (Figure 1). Diameter of each femur was measured and the average value recorded prior to analysis. The femur was placed on two holders, one at each end with 5 mm apart. The holders are perpendicular to the horizontal axis and the force was applied downward at the specimen midpoint (Figure 2). Force at a rate of 10 mm/sec was applied to the midpoint of the femur diaphysis such that the anterior surface was in compression and the posterior surface in tension until it fractures. The load was increased until the bone breaks.

Cross-sectional area and shape of the femur were assumed constant throughout the test. Force and displacement data recorded during the tests were used in combination with bone geometry parameters to compute mechanical properties which comprises of both extrinsic and intrinsic properties [55]. Graph of stress against strain were plotted. The slope of the stress–strain curve in the elastic region represents Young modulus or modulus of elasticity. The extrinsic parameters (load, displacement and stiffness) measure the whole bone properties, while the intrinsic parameters (stress, strain and Young modulus) measure the bone material.

### Statistical analysis

The data were analysed using Statistical Package for Social Sciences software (SPSS 19.0, Chicago, USA). Firstly, the data was tested for normality using the Kolmogorov-Smirnov test (n = <100). For normally distributed data, the statistical tests used were the analysis of variance (ANOVA), followed by Tukey’s HSD test. For data that was not normally distributed, Kruskal-Wallis and Mann–Whitney tests were used. All the results were expressed as mean ± standard error of the mean (SEM).

## Results

### Maximum load (Max load)

**Figure 3:**
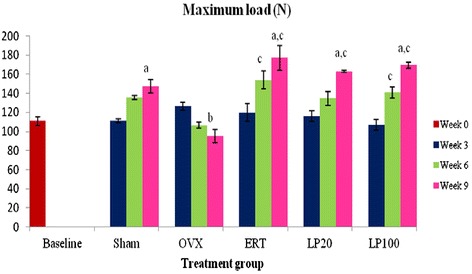
**Maximum load value for all the groups after 3, 6 and 9 weeks of treatment.** Data presented as mean ± SEM (p < 0.05). Sham: sham-operated, OVX: ovariectomized control, ERT: ovariectomized and estrogen supplementation, LP20: ovariectomized with LP supplementation (20 mg/kg), LP100: ovariectomized with LP supplementation (100 mg/kg). ^a^P < 0.05 vs Baseline, ^b^P < 0.05 vs Sham at 9 weeks, ^c^P < 0.05 vs OVX of the corresponding week.

There were gradual increments in the Max load parameter for the Sham, ERT, LP20 and LP100 groups until they were significantly higher than the Baseline group at 9 weeks. At the same period, the Max load parameter of the OVX group were significantly lower than the Sham group. All the treatment groups also showed significantly higher Max load compared to the OVX group (Figure 3).

### Displacement

**Figure 4:**
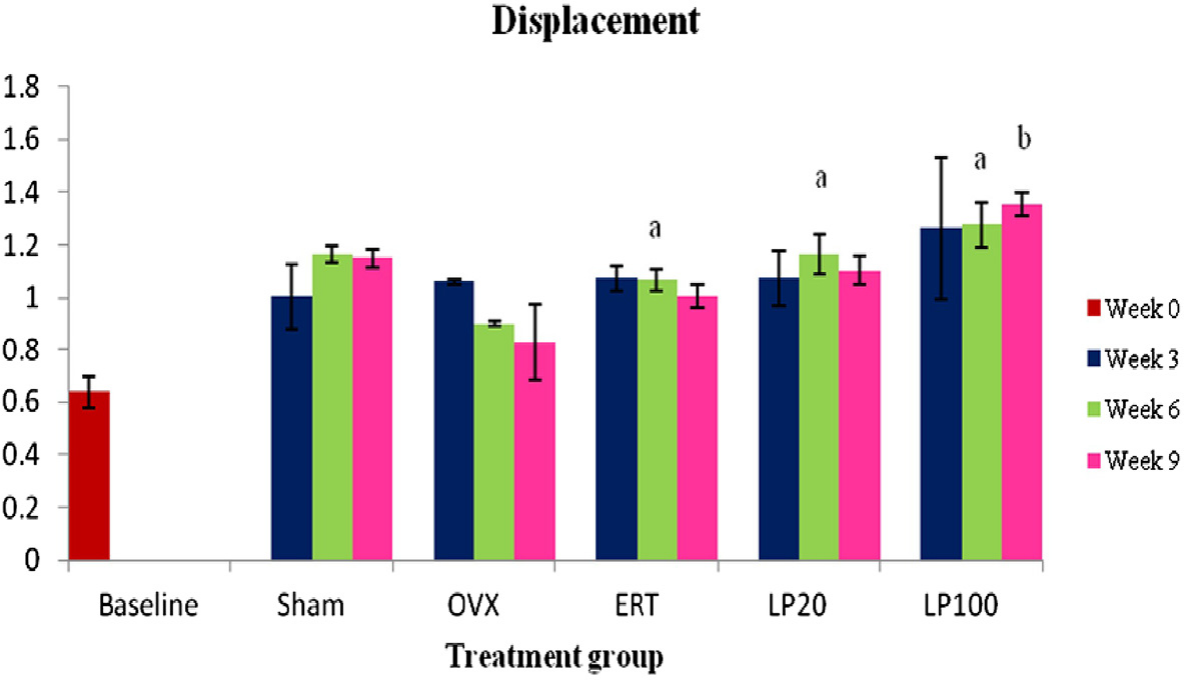
**Displacement value for all the groups after 3, 6 and 9 weeks of treatment.** Data presented as mean ± SEM (p < 0.05). Sham: sham-operated, OVX: ovariectomized control, ERT: ovariectomized and estrogen supplementation, LP20: ovariectomized with LP supplementation (20 mg/kg), LP100: ovariectomized with LP supplementation (100 mg/kg). ^a^P < 0.05 vs OVX at 6 weeks of treatment, ^b^P < 0.05 vs ERT at 9 weeks of treatment.

There was no significant difference in the displacement value between the Sham and OVX groups. At 6 weeks of treatment, all the treatment groups showed significantly higher displacement value than the OVX group at the corresponding treatment duration. At 9 weeks of treatment, the LP100 group showed a significantly higher displacement than the ERT group (Figure 4).

### Stiffness

**Figure 5:**
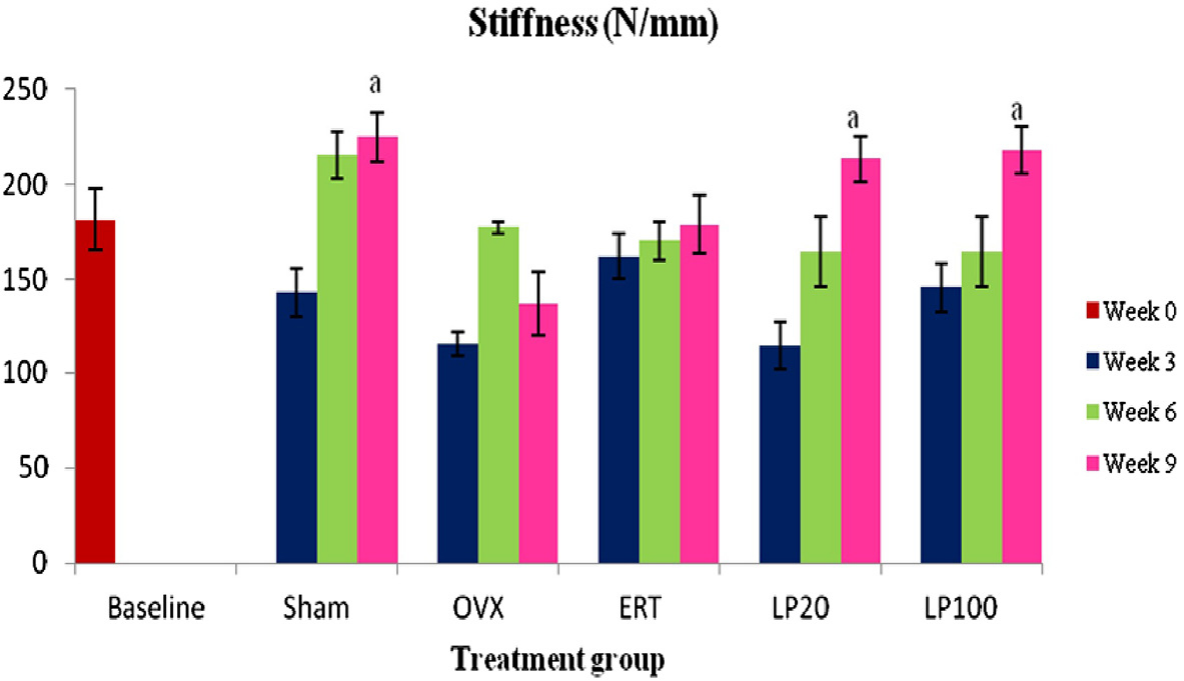
**Stiffness value for all the groups after 3, 6 and 9 weeks of treatment.** Data presented as mean ± SEM (p < 0.05). Sham: sham-operated, OVX: ovariectomized control, ERT: ovariectomized and estrogen supplementation, LP20: ovariectomized with LP supplementation (20 mg/kg), LP100: ovariectomized with LP supplementation (100 mg/kg). ^a^P < 0.05 vs OVX.

An increasing trend in the stiffness value with time can be seen in all the groups except for the OVX group. At 9 weeks of treatment, the Sham, LP20 and LP100 groups showed significantly higher stiffness value than the OVX group (Figure 5).

### Stress

**Figure 6:**
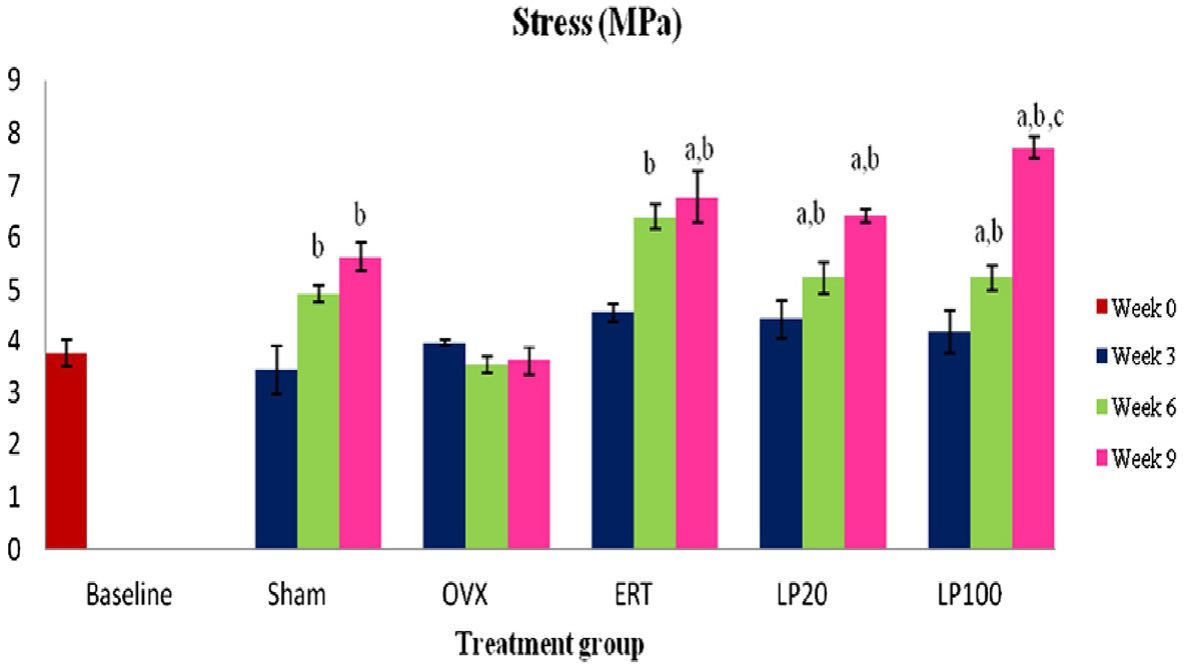
**Stress value for all groups at 3, 6 and 9 weeks of treatment.** Data presented as mean ± SEM (p < 0.05). Sham: sham-operated, OVX: ovariectomized control, ERT: ovariectomized and estrogen supplementation, LP20: ovariectomized with LP supplementation (20 mg/kg), LP100: ovariectomized with LP supplementation (100 mg/kg). ^a^P < 0.05 vs Baseline, ^b^P < 0.05 vs OVX, ^c^P < 0.05 vs ERT.

An increasing trend in the stress value with time can be seen in all the groups except for the OVX group. At 9 weeks of treatment, the ERT and LP-treatment groups showed significantly higher stress value than the Baseline group. At 6 and 9 weeks of treatments, the Sham and all the treatment groups showed significantly higher stress value than the OVX group of the corresponding treatment durations. The stress value of the LP100 group was significantly higher than that of the ERT group (Figure 6).

### Strain

**Figure 7:**
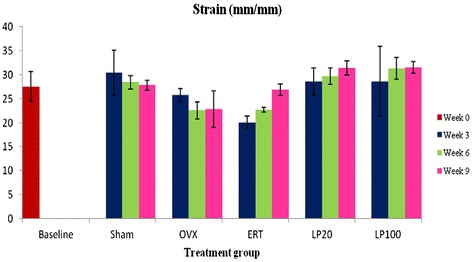
**Strain value for all the groups at 3, 6 and 9 weeks of treatment.** Data presented as mean ± SEM (p < 0.05). Sham: sham-operated, OVX: ovariectomized control, ERT: ovariectomized and estrogen supplementation, LP20: ovariectomized with LP supplementation (20 mg/kg), LP100: ovariectomized with LP supplementation (100 mg/kg).

There was an increasing trend in the strain value for all the treatment groups but no significant difference was reported (Figure 7).

### Young modulus

**Figure 8:**
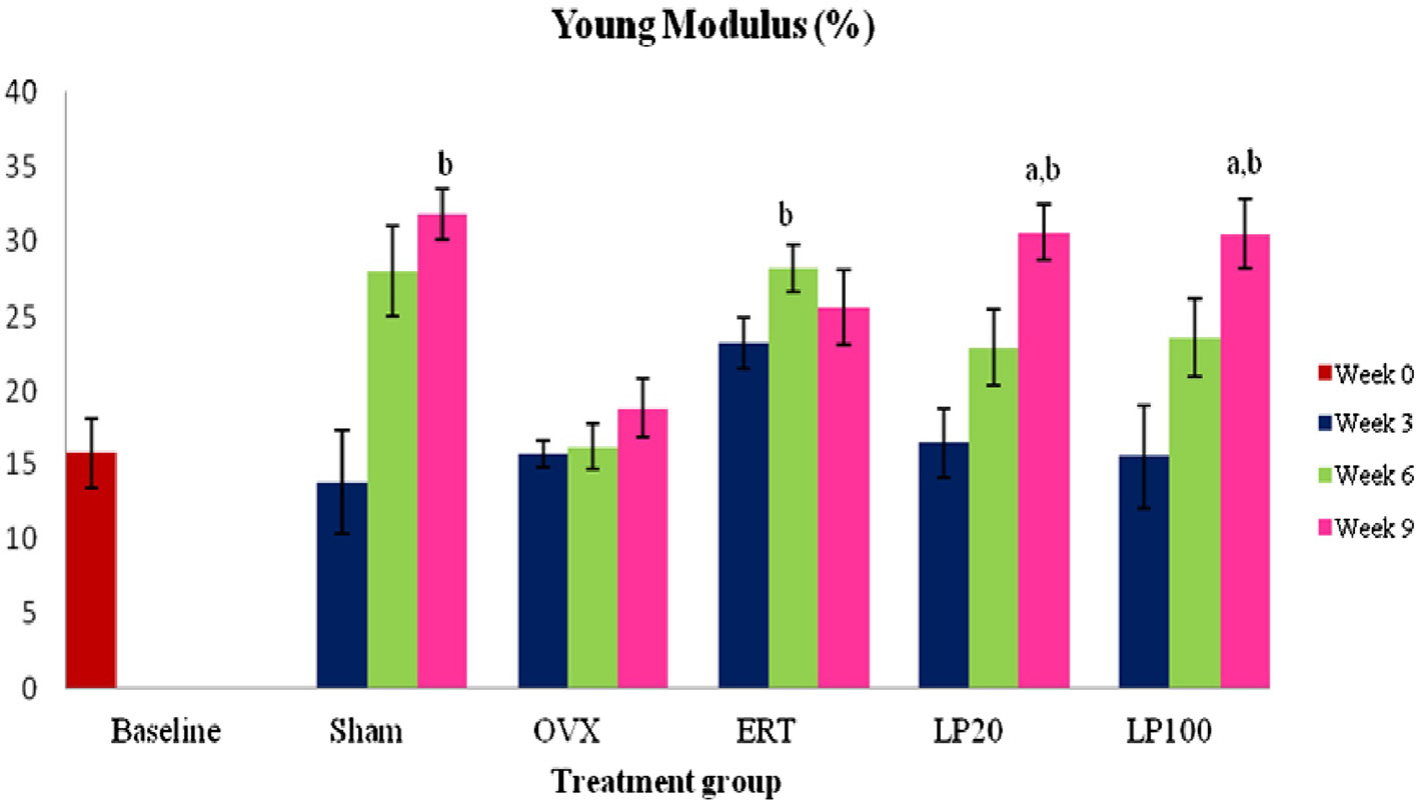
**Young modulus value for all groups after 3, 6 and 9 weeks of treatment.** Data presented as mean ± SEM (p < 0.05). Sham: sham-operated, OVX: ovariectomized control, ERT: ovariectomized and estrogen supplementation, LP20: ovariectomized with LP supplementation at the dose of 20 mg/kg, LP100: ovariectomized with LP supplementation at the dose of 100 mg/kg. ^a^P < 0.05 vs baseline, ^b^P < 0.05 vs OVX.

The Young Modulus of the Sham group was significantly higher than the OVX group at 9 weeks of treatment. There was a time-dependent increment in the Young modulus value for the Sham and LP-treated groups. At 9 weeks of treatment, both the LP20 and LP100 groups showed significantly higher Young modulus than the Baseline and OVX group (Figure 8).

## Discussion

Osteoporosis is one of the most common diseases affecting 1 in 3 women and 1 in 12 men [56]. Worldwide, approximately 200 million women suffered from osteoporosis and by the age of 50 the lifetime risk of osteoporotic fracture is nearly 40% [57]. Although effective treatments are available for osteoporosis, their prolonged use was associated with adverse effects such as breast cancer, thromboembolic and coronary heart disease. This has led to an increase in the demand for alternative medicine to treat and prevent osteoporosis. It has also attracted studies to search for potential agents to replace ERT. In recent years, a medicinal plant known as *Labisia pumila* (LP) was reported to possess anti-osteoporosis activities. It was reported that LP was able to increase bone formation and reduce the bone resorption markers in ovariectomized rats [47]. LP supplementation was also shown to protect the bone structure changes of estrogen-deficient rats [48].

In a previous study, the authors have investigated the time-dependent effects of two doses of LP (20 mg a/kg and 100 mg/kg) using *in vitro* micro-CT. Supplementation of LP 100 mg/kg for 9 weeks showed the best effect in reversing the ovariectomy-induced bone changes. Bone structural parameters such as bone volume fraction, connectivity density and trabecular number were increased and trabecular separation decreased significantly in OVX rats treated with LP100 for 9 weeks. Three dimensional (3D) analysis also showed that the trabecular microarchitecture of LP treated rats improved significantly compared to other groups [58]. Following these positive results of LP on bone microarchitecture, our present study was performed to evaluate further the effects of LP on bone strength.

The diagnosis of osteoporosis is often based on bone density. However, a denser bone does not always mean stronger bone as in the case of fluoride treatment of osteoporosis, where the newly formed bone was weak although it appeared to be dense [59,60]. Hence, it is important to study the bone mass, structure and strength as a whole to determine bone quality. Bone markers and histomorphometry parameters alone are not strong indicators of bone quality. It is the bone strength which plays a vital role in predicting early fracture risk [61,62]. The term bone quality refers to all characteristics beyond bone mass that influence the ability of bone to bear loads [63]. In accordance with this, bone biomechanics should be measured to study its strength. Bone strength analysis may also provide information on the effects of different treatments on bone.

Estrogen deficiency following ovariectomy resulted in tremendous bone loss with bone resorption activity outweighing bone formation activity [64,65]. These bone changes have led to profound reductions in bone density and bone mechanical strength [66]. The trabecular thinning and reduction of horizontal trabeculae resulted in significant reduction in bone stiffness [67,68]. This is in line with Kennedy et al. [69] which reported that ovariectomised rats had reduced stiffness and yield strength. Estrogen maintained bone strength by promoting osteoblast differentiation [70-72] and stimulating the production of osteoprotegrin (OPG), a potent anti-osteoclastogenic factor [73,74]. Estrogen may also increase the anti-oxidative enzyme levels, thus alleviating the oxidative stress-induced bone loss [75].

In this study, two doses of LP were given to ovariectomized rats for three different time intervals; 3, 6 and 9 weeks to evaluate the dose and time-dependent effects of LP on bone biomechanical strength. The 20 mg/kg dose was a round up value of the 17.5 mg/kg standard dose used in a previous study [47,48]. While, the higher dose was set at five times higher to 100 mg/kg. Ideally, several doses of LP should have been tested, but due to ethical reasons, this was not possible as we are required to reduce the number of animals used in the study. According to previous toxicity studies, LP extract was shown to be safe with the lethal dose 50 (LD_50_) of more than 5.0 g/kg [76]. In other studies, LP extract was shown to exhibit no-adverse-effect-level (NOAEL) at the dose of 50 mg/kg in a sub-acute study [77], 1000 mg/kg in a sub-chronic study [78] and 800 mg/kg in reproductive toxicity testing. Therefore, the doses of 20 mg/kg and 100 mg/kg used in the study were safe. In humans, LP is normally taken by women in the dose of 500 to 1000 mg/daily.

Whole bone biomechanical tests can be performed in bending, tension, compression and torsional loading settings [79]. Bending is most commonly applied to rodents’ long bones due to difficulties of machining tensile or compressive specimens from small bones. There are two types of parameters which can be derived from a biomechanical test; the extrinsic and intrinsic parameters. Extrinsic parameters reflect the properties of whole bone which are affected by various external factors. They comprise of load, displacement and stiffness. Intrinsic parameters on the other hand reflect the inner material of bones such as its geometric distribution and cellular metabolic activity affecting the bone’s ability to bear loads. Intrinsic parameters comprise of stress, strain and modulus of elasticity.

In this study, the bone of rats supplemented with LP showed significant improvement in both the extrinsic and intrinsic parameters. As expected, the OVX group showed deterioration in the biomechanical strength parameters and by the ninth-week of treatment most of these parameters were significantly lower than the Sham group. This finding was supported by previous studies which reported that ovariectomized rats showed significant deteriorations in the bone structure in the first three months [80]. Another study reported that there was no sign of bone changes in less than one month after ovariectomy. This is consistent with our findings that there was no significant change in bone strength parameters at 3 weeks post-ovariectomy.

Maximum or ultimate load indicates the whole bone strength at the point where the femur started to change from elastic to plastic phase. Maximum load can be defined as the maximum amount of force needed to break the bone. It reflects the general integrity of the bone structure. The bones of all the treatment groups showed gradual increments in the maximum load until they were significantly higher compared to the Baseline and OVX groups at nine weeks post-ovariectomy. Both doses of LP supplementations improved bone strength and were comparable to ERT in withstanding the given load. Displacement is another extrinsic parameter which is defined as the length of deformation that the bone can sustain before failing [81]. It can be used to measure bone ductility and is inversely related to the brittleness of the bone [82]. Rats supplemented with 100 mg/kg dose of LP for the duration of nine weeks had the most ductile bones compared to others. Their bones were also having significantly higher displacement values compared to the ERT group. These results showed that although both the ERT and LP100 groups had strong bones in terms of sustaining high amount of load, the bones of LP100 were more ductile and hence harder to break.

The load - displacement curve may give a clearer understanding of bone strength. The linear region of this curve represents the elastic property of a bone, where the deformation upon loading is reversible [83]. The gradient under this elastic region depicts the extrinsic stiffness or rigidity. It represents bone mineralization of the relative hydroxyapatite and collagen fibers proportion [84,85]. Beyond the point of yielding is the plastic region, where permanent deformation occurs upon compressive force. In this study, the Sham, LP20 and LP100 groups showed significantly higher bone stiffness at 9 weeks post-treatment compared to the OVX group. The inferior bone stiffness of the OVX group was expected as many studies have reported that ovariectomy affected not only the bone mass but the bone quality as well [86,87]. Surprisingly, LP supplementation was able to improve the bone stiffness of ovariectomised rats while ERT failed to do so.

Bone biomechanical testing is not only focused on the mechanical behaviour of the whole bone but also the mechanical properties at tissue level or intrinsic properties. Intrinsic parameters measured were stress, strain and Young’s modulus. Stress is the strength of the bone tissue under a given loading condition. In this study, the Sham and all the treatment groups were able to receive higher stress compared to the OVX group after 6 and 9 weeks of treatment. The LP100 group was able to receive the greatest stress at 9 weeks, which was significantly higher than the ERT group. This indicated that the bone tissue of the LP100 group was able to absorb higher energy before failure than the ERT group. As for the Strain parameter, it represents ductility of the bone [88,89]. However, there were no significant changes in this parameter for all the groups.

The slope from stress - strain curve represented the modulus of elasticity which is also known as Young’s modulus. Young’s modulus is influenced by the amount of collagen and calcification process in the bone. After 9 weeks of treatment, the Sham, LP20 and LP100 groups had significantly higher bone elasticity than the OVX group. This indicated that the bones of the Sham and LP groups were much more elastic than the OVX group and less likely to fracture. Based on all the results, supplementation of LP at 100 mg/kg were able to preserve bone strength from the deleterious effects of ovariectomy. At this dose, LP was found to be better than ERT in maintaining bone strength and ductility.

There are several possible mechanisms behind the ability of LP to retain bone strength during estrogen deficiency state. LP extract contains triterpenes and saponins, which are known phytoestrogens [90]. Phytoestrogen can mimic or modulate the action of endogenous estrogens by binding to estrogen receptors [91,92]. It was reported that phytoestrogen exerted bone sparing effects in a rat model [93,94]. Similar to estrogen, LP through its phytoestogenic activity may induce and inhibit osteoclasts and osteoblast apoptosis respectively. Therefore, LP may maintain bone strength by reducing bone resorption and increasing bone formation activities [95].

Besides the phytoestrogenic property, LP may exert anti-oxidative effects on the bone. According to Norhaiza et al. [96], the anti-oxidative property of LP was contributed by its content of flavonoids, ascorbic acids, beta-carotene, anthocyanin and phenolic compounds. Estrogen deficiency leads to deterioration in anti-oxidant defense system and upregulation of reactive oxygen species (ROS). These imbalances resulted in lipid peroxidation and bone loss [97]. Hence, supplementation of LP may abate oxidative stress, thus preventing bone loss and maintaining bone strength.

There are some raised concerns on the risk of endometrial hyperplasia and excessive cell growth in the uterus, secondary to the use of phytoestrogens. However, many previous studies had shown that LP exhibits anti-proliferative effects [98]. A polar solvent extract such as LP water extract had been shown to reduce risks of cell proliferation. The bioactive compounds that are responsible for estrogenic activity are more polar in nature hence they are highly expressed in water extract. This is in contrast to a less polar solvent extracts such as ethanol and dichloromethane LP extracts which may induce an increase in cell proliferation [99]. Hence, LP water extract used in this study may exhibit an estrogenic effect without the risk of excessive cell proliferation. This is also supported by previous toxicity studies which reported that LP extract did not alter the rats’ general health and no gross visceral changes of the ovaries or uterus were found at the dose up to 800 mg/kg/day [76,77]. Although LP is safe and effective, further studies are warranted to document a conclusive mechanisms of its therapeutic action.

## Conclusions

As a conclusion, LP supplementation at the dose of 100 mg/kg for 9 weeks duration of treatment was found to be more effective than ERT in maintaining the bone strength of a postmenopausal osteoporosis rat model. Based on its safety profile and ability to preserve bone strength, LP has potential as an alternative treatment for postmenopausal osteoporosis.
